# More than just immaturity: evidence supporting the hypothesis that sleep spindle characteristics reflect GABAergic depolarization in infancy

**DOI:** 10.5935/1984-0063.20220079

**Published:** 2022

**Authors:** Dmitry Chegodaev, Polina Pavlova, Sergey Kiselev

**Affiliations:** Ural Institute of Humanities, Ural Federal University named after the first President of Russia B. N. Yeltsin, Laboratory for Brain and Neurocognitive Development, Department of Psychology - Yekaterinburg - Ural - Russia

**Keywords:** Electroencephalography, Sleep, Infant, GABA-A Receptor Agonists

## Abstract

Sleep spindles are thalamocortical oscillations with waxing-waning morphology, which comprise the key electroencephalographic (EEG) hallmark of stage 2 non-rapid eye movement sleep. The functional role of sleep spindles is not sufficiently clear, but there is a large body of literature that indicates the relationship between spindle activity and neural plasticity. Many of the spindle parameters (frequency, configuration, duration, density, and topography) vary significantly throughout life. However, the long duration, asynchrony and sharp morphology are the most distinctive characteristics of sleep spindles in infants. This unique infantile phenotype of sleep spindles typically changes after approximately one year of postnatal life in humans. Considering that EEG reflects brain electrochemical activity, there is evidence to suggest that substantial neurochemical events underlie these changes. In this paper, we hypothesize that the GABA (gamma-aminobutyric acid) shift is a key event influencing the sleep spindle phenotype during infancy. We briefly review evidence for the relation between infantile sleep spindles and depolarizing GABA transmission occurring in the developing brain.

## INTRODUCTION

According to the American Academy of Sleep Medicine, the definition of sleep spindles is as follows: *“a train of distinct waves with frequency 11-16Hz (most commonly 12-14Hz) with a duration ≥0.5 seconds, usually maximal in amplitude using central derivations”*^1^.

Sleep spindles emerge from thalamocortical interactions and are considered an electroencephalographic hallmark of non-rapid eye movement sleep throughout life. There are two types of sleep spindles that differ in frequency and localization: “slow” spindles (<12Hz) that show maximal distribution over prefrontal cortical areas and “fast” spindles (>12Hz) that are typically registered over parietal and central regions^2^.

In typically developing infants, spindles can be registered as early as 1 month of age, and it was proposed that they could appear earlier in premature infants^3^.

It is well known that characteristics of sleep spindles (such as frequency, duration, density, and synchronicity) are undergoing significant transformation during lifespan and especially during infancy^4,5^ as well as their correlation with brain maturation^6^. In particular, asynchronous sleep spindles, which are observed in children up to 2 years old, can serve as a marker of incomplete myelination of the corpus callosum^7^.

The frequency during the second half of the first year of life shows a temporary tendency to decrease from 13-13.5Hz (at 2-6 months of age) to 12Hz at approximately 12 months of age^8,9^. However, to our knowledge, morphological characteristics are rather poorly discussed in the literature.

The fact that sleep spindles in infants have distinctive morphological features was first described in 1961 by Fois (1961)^10^. The waves in its structure are diphasic: they consist of a positive sharp component and a negative rounded (or smooth) component^11^. After the age of 1 year, the spindles appear to be monophasic^12^. This has led to appearance-specific epithets such as “sharp spindles of infancy” or “mu-like sleep spindles”, highlighting their visual similarities with mu rhythm. Another expressive feature of sleep spindles in infancy is their long duration (up to 15-20 seconds)^5,8^. The long duration of spindles is considered a rather strong developmental marker that is typical for infants up to six months of age and disappears in the second half of the first year of life^12,13^.

Thus, the characteristics of sleep spindles in infants are well described. However, the mechanisms determining these parameters are poorly understood.

Here we briefly review possible neurochemical aspects of these infantile spindle characteristics and support the hypothesis implying a link between them, the depolarizing action of GABA (γ-aminobutyric acid) and the GABA shift.

## THE HYPOTHESIS

One of the most critical neurochemical events occurring in the brain during early postnatal development is the so-called GABA shift. Briefly, the GABA shift is an evolutionarily conserved switch from excitatory to inhibitory neurotransmission (from depolarizing to hyperpolarizing GABAergic action) that depends on the altered expression of chloride transporters: sodium-potassium-chloride cotransporter 1 (NKCC1) and potassium-chloride cotransporter 2 (KCC2)^14,15^. The postnatal GABA shift is the final shift in a sequence of GABA shifts, regulating the proliferation, migration, differentiation of neurons, and synaptogenesis^14,16^.

We are of the opinion that this key neurochemical process behind brain maturation should be reflected in noninvasive electrophysiological recordings (EEG). The main assumption of this work is as follows: the change of morphology (i.e., loss of diphasic configuration) of sleep spindles and decreasing spindle duration during infancy might reflect a GABA shift at the neurochemical level.

## EARLY GABA-MEDIATED DEPOLARIZATION CONTRIBUTES TO THE CONFIGURATION SHARP SPINDLES OF INFANCY

Roughly speaking, the term “sharpness” in electrophysiology is associated with depolarization. Depolarization is classically defined as a process caused by the influx of Na^+^ ions into a neuron through the opening of voltage-gated Na^+^ channels. Although GABAergic depolarization is apparently independent of voltage-gated Na^+^ channels^17^, it is pronounced and significantly contributes to the depolarization observed in the developing brain.

As mentioned above, early in development, GABAergic transmission changes to an inhibitory fashion during the first postnatal year.

The depolarizing action of GABA in the brain during early infancy is a result of a high intracellular concentration of chloride caused by high expression of NKCC1 and significantly lower expression of KCC1. GABAA receptor activation causes Cl^-^ efflux and depolarization. Under these conditions, activation of γ-aminobutyric acid type A (GABAA) receptors leads to chloride efflux and depolarization.

It is generally accepted that GABA and glutamate signaling are interconnected. Therefore, it is not surprising that GABAergic depolarization could facilitate glutamatergic-signaling^18^. Activation of GABAA receptors removes the magnesium block of N-methyl-D-aspartate (NMDA) receptors, which in turn leads to an increase in calcium (Ca2^+^) influx and further enhances the depolarizing effect of GABA^19,20^.

In the developing rodent cortex, NKCC1 expression is maximal during postnatal day (P) 3-P7 and then significantly decreases after P14. At the same time, KCC2 expression was low during the first postnatal week and demonstrated an increase at P14-15. The similar expression pattern (i.e., high neuronal expression of NKCC1 and low expression of KCC2 in neurons during early infancy) are present in humans before the end of the first year of life. Dzhala et al. (2005)^21^ showed that NKCC1 expression was significantly higher than that at 1 year and older. During the first year of life (until 92 weeks postconceptial age), NKCC1 expression rapidly decreased to levels of the adult. In contrast, KCC1 expression shows minimal levels when NKCC1 levels peak and reaches adult-like levels after the first year of life^21,22^.

It is important to note that the period from P7 to P14 in rodents approximately corresponds to the same stage of brain development as human infants between term birth and the first year of life^23^.

Taking into account that Ca^2+^-dependent small-conductance-type 2 (SK2) K^+^ channels underlie spindle generation^24^, it is also possible that these channels contribute to the sharpness of spindle waves. SK channels are a group of ion channels that are activated solely by intracellular Ca^2+^. Activation of SK channels (primarily SK2) mediates medium after hyperpolarization and reduces the firing frequency of action potentials; thus, SK2 channels play a critical role in neuronal excitability^25,26^.

Decreasing SK channel activity, obviously as well as their low expression levels, leads to enhanced neuronal excitability^24^. Interestingly, these channels show expressional trajectories similar to GABAergic system maturation in the brain. SK2 channels are characterized by low expression during early development and significantly increase during the first year of life. In rodents, SK2 expression revealed a 3-fold increase before P15 and then followed a plateau^27^.

## GABA SHIFT INFLUENCES ON DURATION OF SLEEP SPINDLES

The fact that the GABA shift is a crucial factor regulating cortical development is widely recognized. As mentioned above, the depolarizing actions of GABA regulate neurogenesis, especially synaptogenesis and synapse maturation, which remain crucial processes for cortical development after birth.

The increasing expression of KCC2 (more precisely KCC2b isoform), together with the parallel reduction of NKCC1, in the cortex during early postnatal development is associated with the formation and maturation of excitatory synapses in the cortex. In particular, the developmental expression of KCC2 showed a strong parallel to synaptophysin, routinely used as a marker reflecting the density of glutamatergic synapses^28^.

Recent studies have shown that cortical neurons begin to express NMDA receptors early in development, but glutamatergic synapses remain inactive (or “silent”) due to the blockade of the receptors by Mg^2+^ ions^29^, which is gradually decreased as a result of GABAergic depolarization (see above). During early postnatal development, a vast majority of thalamocortical synapses is “silent”, and they are finally converted into functional synapses by postnatal day 15 in rats^30^. Thus, by the time a GABA shift, the neocortex achieves sufficient functional maturation to support effective corticothalamic feedback. These results are consistent with Bonjean et al. (2011)^31^ and further support the idea that neocortical feedback can mediate spindle termination.

Interestingly, the GABA shift demonstrates regional and age-specific differences^32,33^. In other words, shift does not occur at the same time point in the whole cortex but depends on the maturational course of certain cortical regions. This fact, in turn, highlights the link between GABA excitatory action, GABA excitatory/inhibitory shift and synapse formation^18,34^.

## CONCLUDING REMARKS AND FUTURE PERSPECTIVES

In view of the foregoing, we argue that at least two characteristics of sleep spindles in infants may be associated with GABA depolarization. Their alterations coincide with the postnatal switch of GABA transmission from excitatory to inhibitory.

The reduction in spindle duration indirectly reflects the GABA switch. This event is largely determined by the maturation of cortical synapses, which leads to strengthening of corticothalamic feedback, mediating spindle termination. Apparently, corticothalamic feedback reaches the necessary stage of maturity before the full completion of the GABA shift, which would explain the fact that a long spindle duration is rarely observed in infants after six months of age.

However, a sharp configuration reflects persisting GABAergic depolarization in the brain until the full completion of the GABA shift after 1 year of age in humans (at approximately 12-13 months to our understanding). One intriguing question is why some EEG patterns observed during adulthood (mu rhythm and midline theta [Ciganek] rhythm) have a similar configuration to infantile sleep spindles. The arguments from this paper imply that the morphology of mu rhythm and Ciganek rhythm are also determined by GABA-depolarizing action. It should be noted that these EEG patterns are characterized by similar topography (over the sensorimotor cortex). Lee et al. (2012)^35^ shed some light on this question. Their study^35^ demonstrated that GABA can rapidly switch from hyperpolarization to depolarization in adult sensorimotor cortical neurons.

The evidence from their study indicates that some unique features of organization of the sensorimotor cortex are determinative factors for the GABA switch in adults.

In conclusion, it is well known that a depolarizing action of GABA during early postnatal development determines increased susceptibility of the neonatal brain to seizures and low effectiveness of antiepileptic drugs, mediating its anticonvulsant action by enhancing the action of GABA at GABA-A receptors^36^.

Considering all the above-mentioned factors, we believe that immature spindle characteristics (especially at nontypical ages) can be considered a simple routine marker of possible ineffectiveness of GABA-A receptor agonists in children with epilepsy.

It will also be interesting to investigate the diagnostic potential of long persisting infantile spindles in diseases associated with defects in GABA shift, such as MeCP2-related diseases and Fragile X syndrome.

However, further studies will be needed for an accurate elucidation of these issues.

## References

[1] Iber C, Ancoli-Israel S, Chesson A, Quan SF (2007). The AASM manual for the
scoring of sleep and associated events: rules. Terminology and technical
specifications.

[2] Mölle M, Bergmann TO, Marshall L, Born J (2011). Fast and slow spindles during the sleep slow oscillation:
disparate coalescence and engagement in memory processing. Sleep.

[3] Gruber R, Wise MS (2016). Sleep spindle characteristics in children with neurodevelopmental
disorders and their relation to cognition. Neural Plast.

[4] Clawson BC, Durkin J, Aton SJ (2016). Form and function of sleep spindles across the
lifespan. Neural Plast.

[5] Louis J, Zhang JX, Revol M, Debilly G, Challamel MJ (1992). Ontogenesis of nocturnal organization of sleep spindles: a
longitudinal study during the first 6 months of life. Electroencephalogr Clin Neurophysiol.

[6] McClain IJ, Lustenberger C, Achermann P, Lassonde JM, Kurth S, LeBourgeois MK (2016). Developmental changes in sleep spindle characteristics and sigma
power across early childhood. Neural Plast.

[7] Shahid A (2021). Electroencephalogram and pitfalls in children: Benign variants
and how they can be misinterpreted. Karnataka Pediatr J.

[8] Dan B, Boyd SG (2006). A neurophysiological perspective on sleep and its
maturation. Dev Med Child Neurol.

[9] Sokoloff G, Dooley JC, Glanz RM, Wen RY, Hickerson MM, Evans LG (2021). Twitches emerge postnatally during quiet sleep in human infants
and are synchronized with sleep spindles. Curr Biol.

[10] Fois A (1961). The electroencephalogram of the normal child.

[11] Schomer D, Silva FL (2018). Niedermeyer’s electroencephalography: basic principles, clinical
applications, and related fields.

[12] Jankel WR, Niedermeyer E (1985). Sleep spindles. J Clin Neurophysiol.

[13] Hughes JR (1985). Sleep Spindles revisited. J Clin Neurophysiol.

[14] Peerboom C, Wierenga CJ (2021). The postnatal GABA shift: a developmental
perspective. Neurosci Biobehav Rev.

[15] Leterrier C (2020). GABA in, garbage out: AIS-located proteasomes regulate the
developmental GABA switch. J Cell Biol.

[16] Oh WC, Lutzu S, Castillo PE, Kwon HB (2016). De novo synaptogenesis induced by GABA in the developing mouse
cortex. Science.

[17] Kirmse K, Kummer M, Kovalchuk Y, Witte OW, Garaschuk O, Holthoff K (2015). GABA depolarizes immature neurons and inhibits network activity
in the neonatal neocortex in vivo. Nat Commun.

[18] Basu SK, Pradhan S, du Plessis AJ, Ben-Ari Y, Limperopoulos C (2021). GABA and glutamate in the preterm neonatal brain: In vivo
measurement by magnetic resonance spectroscopy. Neuroimage.

[19] Wang DD, Kriegstein AR (2008). GABA regulates excitatory synapse formation in the neocortex via
NMDA receptor activation. J Neurosci.

[20] Leinekugel X, Medina I, Khalilov I, Ben-Ari Y, Khazipov R (1997). Ca2+ oscillations mediated by the synergistic excitatory actions
of GABA(A) and NMDA receptors in the neonatal hippocampus. Neuron.

[21] Dzhala VI, Talos DM, Sdrulla DA, Brumback AC, Mathews GC, Benke TA (2005). NKCC1 transporter facilitates seizures in the developing
brain. Nat Med.

[22] Schulte JT, Wierenga CJ, Bruining H (2018). Chloride transporters and GABA polarity in developmental,
neurological and psychiatric conditions. Neurosci Biobehav Rev.

[23] Stafstrom CE, Velisek L (2019). New.

[24] Wimmer RD, Astori S, Bond CT, Rovó Z, Chatton JY, Adelman JP (2012). Sustaining sleep spindles through enhanced SK2-channel activity
consolidates sleep and elevates arousal threshold. J Neurosci.

[25] Nam YW, Baskoylu SN, Gazgalis D, Orfali R, Cui M, Hart AC (2018). A V-to-F substitution in SK2 channels causes Ca2+
hypersensitivity and improves locomotion in a C. elegans ALS
model. Sci Rep.

[26] Villalobos C, Shakkottai VG, Chandy KG, Michelhaugh SK, Andrade R (2004). SKCa channels mediate the medium but not the slow
calcium-activated after hyperpolarization in cortical
neurons. J Neurosci.

[27] Ballesteros-Merino C, Lin M, Wu WW, Ferrandiz-Huertas C, Cabañero MJ, Watanabe M (2012). Developmental profile of SK2 channel expression and function in
CA1 neurons. Hippocampus.

[28] Sedmak G, Jovanov-Milošević N, Puskarjov M, Ulamec M, Krušlin B, Kaila K (2016). Developmental expression patterns of KCC2 and functionally
associated molecules in the human brain. Cereb Cortex.

[29] Wang DD, Kriegstein AR (2009). Defining the role of GABA in cortical development. J Physiol.

[30] Meng X, Kao JP, Kanold PO (2014). Differential signaling to subplate neurons by spatially specific
silent synapses in developing auditory cortex. J Neurosci.

[31] Bonjean M, Baker T, Lemieux M, Timofeev I, Sejnowski T, Bazhenov M (2011). Corticothalamic feedback controls sleep spindle duration in
vivo. J Neurosci.

[32] Murata Y, Colonnese MT (2020). GABAergic interneurons excite neonatal hippocampus in
vivo. Sci Adv.

[33] Tyzio R, Holmes GL, Ben-Ari Y, Khazipov R (2007). Timing of the developmental switch in GABA(A) mediated signaling
from excitation to inhibition in CA3 rat hippocampus using gramicidin
perforated patch and extracellular recordings. Epilepsia.

[34] Huttenlocher PR, Dabholkar AS (1997). Regional differences in synaptogenesis in human cerebral
cortex. J Comp Neurol.

[35] Lee J, Woo J, Favorov OV, Tommerdahl M, Lee CJ, Whitsel BL (2012). Columnar distribution of activity dependent gabaergic
depolarization in sensorimotor cortical neurons. Mol Brain.

[36] Khanna A, Walcott BP, Kahle KT (2013). Limitations of current GABA agonists in neonatal seizures: toward
GABA modulation via the targeting of neuronal Cl(-)
transport. Front Neurol.

